# Supplementary material for: Pilots for Healthy and Active Ageing (PHArA-ON) Project: Definition of New Technological Solutions for Older People in Italian Pilot Sites Based on Elicited User Needs
Source: Sensors (Basel). 2021 Dec 27;22(1):163. doi: 10.3390/s22010163 (PMC8749804; doi:10.3390/s22010163)
Supplement: Supplementary file 1 [file sensors-22-00163-s001.zip › sensors-1424024-supplementary.pdf]

Supplementary materials

Table S1. Demographic and clinical characteristics of participants recruited in UP.

	**Older** n = 12	**Informal caregiver** n = 12	**Professional caregiver** n = 8	**Social operators** n = 4	**P value**
**Presence of Physical frailty** – Yes	90%	-	-	-	-
**Presence of Cognitive frailty** – Yes	50%	-	-	-	-
**MMSE** – mean ± sd	25.50 ± 1.17	-	-	-	-
**How long have you been dealing with the elderly with physical and/or cognitive frailty?** (in years)– mean ± sd	-	-	14.40 ± 13.98	-	-
**How long have you been caring for your relative?** (in months) – mean ± sd	-	95.75 ± 120.00	-	-	-
**How many is the time day care?** (in hours) – mean ± sd	-	8.13 ± 9.82	-	-	-
**Sex** – Male/Female<br>Male (%)	5/7<br>41.70	4/8<br>33.30	2/6<br>25.00	0/4<br>0	0.454
**Age** – < 30 – n (%)<br>30-45 – n (%)<br>46-55 – n (%)<br>55-65 – n (%)<br>65-75 – n (%)<br>>75 – n (%)	0 (0)<br>0 (0)<br>0 (0)<br>0 (0)<br>5 (83.3)<br>7 (100)	0 (0)<br>2 (25)<br>4 (80)<br>6 (66.7)<br>0 (0)<br>0 (0)	1 (100)<br>4 (50)<br>0 (0)<br>2 (22.2)<br>1 (16.7)<br>0 (0)	0 (0)<br>2 (25)<br>1 (20)<br>1 (11.1)<br>0 (0)<br>0 (0)	**< 0.0001**
**Educational level** – Elementary school – n (%)<br>Middle school – n (%)<br>High school – n (%)<br>Degree – n (%)	4 (100)<br>4 (44.4)<br>2 (14.3)<br>2 (22.2)	0 (0)<br>3 (33.3)<br>7 (50)<br>2 (22.2)	0 (0)<br>2 (22.2)<br>5 (35.7)<br>1 (11.1)	0 (0)<br>0 (0)<br>0 (0)<br>4 (44.5)	**0.003**

**Table S2**. Demographic and clinical characteristics of participants recruited in CSS.				
	**Older** n = 10	**Informal caregiver** n = 10	**Professional caregiver** n = 5	**P value**
**Presence of Physical frailty** – Yes	0%	-	-	-
**Presence of Cognitive frailty** – Yes	0%	-	-	-
**MMSE** – mean ± sd	28.08 ± 1.18	-	-	-
**How long have you been dealing with the elderly with physical and/or cognitive frailty?** (in years)– mean ± sd	-	-	7.20 ± 6.18	-
**How long have you been caring for your relative?** (in months) – mean ± sd	-	14.90 ± 17.23	-	-
**How many is the time day care?** (in hours) – mean ± sd	-	6.50 ± 6.72	-	-
**Sex** – Male/Female<br>Male (%)	6/4<br>40	7/3<br>30	2/3<br>30	0.535
**Age** – < 30 – n (%)<br>30-45 – n (%)<br>46-55 – n (%)<br>55-65 – n (%)<br>65-75 – n (%)<br>>75 – n (%)	0 (0)<br>0 (0)<br>0 (0)<br>1 (10)<br>7 (70)<br>2 (20)	1 (10)<br>4 (40)<br>5 (50)<br>0 (0)<br>0 (0)<br>0 (0)	1 (20)<br>3 (60)<br>1 (20)<br>0 (0)<br>0 (0)<br>0 (0)	**0.002**
**Educational level** – Elementary school – n (%)<br>Middle school – n (%)<br>High school – n (%)<br>Degree – n (%)	2 (20)<br>3 (30)<br>1 (10)<br>4 (40)	0 (0)<br>0 (0)<br>3 (30)<br>7 (70)	0 (0)<br>0 (0)<br>0 (0)<br>5 (100)	0.069

**Table S3**. Attitudes towards the technologies to be used in the PHArA-ON project, comparing UP and CSS older people feedbacks.			
	**UP** n = 12	**CSS** n = 10	**P value**
**Use of computer/Smartphone/Tablet – Yes**<br>**No**	83.3%<br>16.7%	80.0%<br>20.0%	0.840
**Family/friends use Computer/Smarphone Thablet – Yes**<br>**No**	83.3%<br>16.7%	100.0%<br>0%	0.176
**Technology utility: Robot** – mean ± sd<br>**IoT** – mean ± sd<br>**AI** – mean ± sd<br>**App and Mobile devices** – mean ± sd<br>**WS** – mean ± sd<br>**Virtual/Augmented reality** – mean ± sd	1.92 ± 1.68<br>1.83 ± 1.80<br>2.17 ± 2.16<br>1.33 ± 1.30<br>1.42 ± 1.31<br>2.92 ± 2.68	2.20 ± 1.23<br>2.00 ± 1.54<br>2.50 ± 1.17<br>1.80 ± 1.48<br>1.10 ± 0.32<br>3.60 ± 2.63	0.662<br>0.799<br>0.724<br>0.440<br>0.466<br>0.555
**Technology goals:**			
- **Robot** (Primary function)<br>● *To perform care and assistance tasks – n (%)*	7 (58.3)	4 (40.0)	0.300

• *To help operators in their work – n (%)*	2 (16.7)	4 (40.0)	
• *To improve self-care – n (%)*	2 (16.7)	0 (0)	
• *To have social opportunities – n (%)*	0 (0)	0 (0)	
• *To stay in communication with others – n (%)*	0 (0)	0 (0)	
• *To help family members – n (%)*	1 (8.3)	2 (20.0)	
- **Robot** (Secondary function)			
• *To perform care and assistance tasks – n (%)*	1 (8.3)	2 (20.0)	0.308
• *To help operators in their work – n (%)*	5 (41.7)	2 (20.0)	
• *To improve self-care – n (%)*	1 (8.3)	0 (0)	
• *To have social opportunities – n (%)*	0 (0)	1 (10.0)	
• *To stay in communication with others – n (%)*	0 (0)	2 (20)	
• *To help family members – n (%)*	5 (41.7)	3 (30.0)	
- **IoT**(Primary function)			
• *To perform care and assistance tasks – n (%)*	2 (18.2)	0 (0)	0.105
• *To help operators in their work – n (%)*	1 (9.1)	1 (10.0)	
• *To improve self-care – n (%)*	4 (36.4)	1 (10.0)	
• *To have social opportunities – n (%)*	2 (18.2)	1 (10.0)	
• *To stay in communication with others – n (%)*	0 (0)	5 (50.0)	
• *To help family members – n (%)*	2 (18.2)	2 (20.0)	
- **IoT**(Secondary function)			
• *To perform care and assistance tasks – n (%)*	2 (16.7)	0 (0)	0.551
• *To help operators in their work – n (%)*	2 (16.7)	3 (30.0)	
• *To improve self-care – n (%)*	0 (0)	0 (0)	
• *To have social opportunities – n (%)*	1 (8.3)	2 (20.0)	
• *To stay in communication with others – n (%)*	3 (25.0)	3 (30.0)	
• *To help family members – n (%)*	4 (33.3)	2 (20.0)	
- **AI** (Primary function)			
• *To perform care and assistance tasks – n (%)*	5 (41.5)	3 (30.0)	0.286
• *To help operators in their work – n (%)*	4 (33.3)	3 (30.0)	
• *To improve self-care – n (%)*	1 (8.3)	0 (0)	
• *To have social opportunities – n (%)*	0 (0)	0 (0)	
• *To stay in communication with others – n (%)*	0 (0)	2 (20.0)	
• *To help family members – n (%)*	1 (16.7)	2 (20.0)	
- **AI** (Secondary function)			
• *To perform care and assistance tasks – n (%)*	6 (50.0)	0 (0)	0.055
• *To help operators in their work – n (%)*	0 (0)	4 (40.0)	
• *To improve self-care – n (%)*	3 (25.0)	2 (20.0)	
• *To have social opportunities – n (%)*	0 (0)	1 (10.0)	
• *To stay in communication with others – n (%)*	1 (8.3)	0 (0)	
• *To help family members – n (%)*	2 (16.7)	3 (30.0)	
- **App and Mobile devices** (Primary function)			
• *To perform care and assistance tasks – n (%)*	2 (18.2)	1 (10.0)	0.727
• *To help operators in their work – n (%)*	0 (0)	2 (20.0)	
• *To improve self-care – n (%)*	2 (18.2)	1 (10.0)	
• *To have social opportunities – n (%)*	3 (27.3)	3 (30.0)	
• *To stay in communication with others – n (%)*	3 (27.3)	2 (20.0)	
• *To help family members – n (%)*	1 (9.1)	1 (10.0)	
- **App and Mobile devices** (Secondary function)			
• *To perform care and assistance tasks – n (%)*	1 (8.3)	1 (10.0)	0.801
• *To help operators in their work – n (%)*	5 (41.7)	2 (20.0)	
• *To improve self-care – n (%)*	1 (8.3)	0 (0)	

● *To have social opportunities – n (%)*	0 (0)	2 (20.0)	
● *To stay in communication with others – n (%)*	0 (0)	3 (30.0)	
● *To help family members – n (%)*	5 (41.7)	2 (20.0)	
- **WS** (Primary function)			
● *To perform care and assistance tasks – n (%)*	2 (18.2)	1 (10.0)	0.373
● *To help operators in their work – n (%)*	1 (9.1)	4 (40.0)	
● *To improve self-care – n (%)*	4 (36.4)	4 (40.0)	
● *To have social opportunities – n (%)*	2 (18.2)	0 (0)	
● *To stay in communication with others – n (%)*	0 (0)	0 (0)	
● *To help family members – n (%)*	2 (18.2)	1 (10.0)	
- **WS** (Secondary function)			
● *To perform care and assistance tasks – n (%)*	2 (16.7)	1 (10.0)	0.359
● *To help operators in their work – n (%)*	2 (16.7)	3 (30.0)	
● *To improve self-care – n (%)*	0 (0)	1 (10.0)	
● *To have social opportunities – n (%)*	1 (8.3)	1 (10.0)	
● *To stay in communication with others – n (%)*	3 (25.0)	1 (10.0)	
● *To help family members – n (%)*	4 (33.3)	3 (30.0)	
- **Virtual/Augmented reality** (Primary function)			
● *To perform care and assistance tasks – n (%)*	7 (58.3)	2 (20.0)	0.122
● *To help operators in their work – n (%)*	2 (16.7)	4 (40.0)	
● *To improve self-care – n (%)*	2 (16.7)	3 (30.0)	
● *To have social opportunities – n (%)*	0 (0)	1 (10.0)	
● *To stay in communication with others – n (%)*	0 (0)	0 (0)	
● *To help family members – n (%)*	1 (8.3)	0 (0)	
- **Virtual/Augmented reality** (Secondary function)			
● *To perform care and assistance tasks – n (%)*	1 (8.3)	1 (10.0)	0.147
● *To help operators in their work – n (%)*	5 (41.7)	1 (10.0)	
● *To improve self-care – n (%)*	1 (8.3)	0 (0)	
● *To have social opportunities – n (%)*	0 (0)	5 (50.0)	
● *To stay in communication with others – n (%)*	0 (0)	2 (20.0)	
● *To help family members – n (%)*	5 (41.7)	1 (10.0)	

Table S4. Attitudes towards the technologies to be used in the PHArA-ON project, comparing UP and CSS caregiver feedbacks.

	**UP** n = 22	**CSS** n = 15	**P value**
**Use of computer/Smartphone/Tablet – Yes**	100%	100%	-
**No**	0%	0%	
**Family/friends use Computer/Smarphone Thablet – Yes**	100%	100%	-
**No**	0%	0%	
**Technology utility: Robot** – mean ± sd	2.68 ± 1.98	2.07 ± 1.03	0.279
**IoT** – mean ± sd	1.95 ± 1.79	1.60 ± 0.91	0.484
**AI** – mean ± sd	2.86 ± 2.05	2.13 ± 1.36	0.236
**App and Mobile devices** – mean ± sd	2.05 ± 1.78	2.13 ± 0.74	0.858
**WS** – mean ± sd	1.64 ± 1.96	1.87 ± 1.06	0.682
**Virtual/Augmented reality** – mean ± sd	3.36 ± 2.36	2.80 ± 1.42	0.415
**Technology goals:**			
- **Robot** (Primary function)			
● *To perform care and assistance tasks – n (%)*	10 (52.6)	4 (26.7)	0.521
● *To help operators in their work – n (%)*	6 (31.6)	7 (46.7)	
● *To improve self-care – n (%)*	1 (5.3)	1 (6.7)	
● *To have social opportunities – n (%)*	0 (0)	1 (6.7)	
● *To stay in communication with others – n (%)*	0 (0)	0 (0)	

• *To help family members – n (%)*<br>• *No response – n*	2 (10.5)<br>3	2 (13.3)<br>0	
- **Robot** (Secondary function)<br>• *To perform care and assistance tasks – n (%)*<br>• *To help operators in their work – n (%)*<br>• *To improve self-care – n (%)*<br>• *To have social opportunities – n (%)*<br>• *To stay in communication with others – n (%)*<br>• *To help family members – n (%)*<br>• *No response – n*	 2 (11.1)<br>4 (22.2)<br>1 (5.6)<br>0 (0)<br>5 (27.8)<br>6 (33.3)<br>4	 3 (20.0)<br>4 (26.7)<br>0 (0)<br>0 (0)<br>4 (26.7)<br>4 (26.7)<br>0	 0.835
- **IoT** (Primary function)<br>• *To perform care and assistance tasks – n (%)*<br>• *To help operators in their work – n (%)*<br>• *To improve self-care – n (%)*<br>• *To have social opportunities – n (%)*<br>• *To stay in communication with others – n (%)*<br>• *To help family members – n (%)*<br>• *No response – n*	 4 (20.0)<br>8 (40.0)<br>1 (5.0)<br>3 (15.0)<br>4 (20.0)<br>0 (0)<br>2	 3 (20.0)<br>2 (13.3)<br>4 (26.7)<br>3 (20.0)<br>1 (6.7)<br>2 (13.3)<br>0	 0.117
- **IoT** (Secondary function)<br>• *To perform care and assistance tasks – n (%)*<br>• *To help operators in their work – n (%)*<br>• *To improve self-care – n (%)*<br>• *To have social opportunities – n (%)*<br>• *To stay in communication with others – n (%)*<br>• *To help family members – n (%)*<br>• *No response – n*	 1 (5.6)<br>4 (22.2)<br>3 (16.7)<br>1 (5.6)<br>6 (33.3)<br>3 (16.7)<br>4	 1 (6.7)<br>7 (46.7)<br>0 (0)<br>0 (0)<br>7 (46.7)<br>0 (0)<br>0	 0.174
- **AI** (Primary function)<br>• *To perform care and assistance tasks – n (%)*<br>• *To help operators in their work – n (%)*<br>• *To improve self-care – n (%)*<br>• *To have social opportunities – n (%)*<br>• *To stay in communication with others – n (%)*<br>• *To help family members – n (%)*<br>• *No response – n*	 4 (20.0)<br>8 (40.0)<br>2 (10.0)<br>2 (10.0)<br>2 (10.0)<br>2 (10.0)<br>2	 5 (33.3)<br>3 (20.0)<br>2 (13.3)<br>0 (0)<br>3 (20.0)<br>2 (13.3)<br>0	 0.557
- **AI** (Secondary function)<br>• *To perform care and assistance tasks – n (%)*<br>• *To help operators in their work – n (%)*<br>• *To improve self-care – n (%)*<br>• *To have social opportunities – n (%)*<br>• *To stay in communication with others – n (%)*<br>• *To help family members – n (%)*<br>• *No response – n*	 2 (10.0)<br>3 (15.0)<br>8 (40.0)<br>1 (5.0)<br>1 (5.0)<br>5 (25.0)<br>2	 3 (20.0)<br>3 (20.0)<br>4 (26.7)<br>0 (0)<br>3 (20.0)<br>2 (13.3)<br>0	 0.522
- **App and Mobile devices** (Primary function)<br>• *To perform care and assistance tasks – n (%)*<br>• *To help operators in their work – n (%)*<br>• *To improve self-care – n (%)*<br>• *To have social opportunities – n (%)*<br>• *To stay in communication with others – n (%)*<br>• *To help family members – n (%)*<br>• *No response – n*	 1 (5.3)<br>2 (10.5)<br>5 (26.3)<br>5 (26.3)<br>6 (31.6)<br>0 (0)<br>3	 2 (13.3)<br>0 (0)<br>1 (6.7)<br>4 (26.7)<br>7 (46.7)<br>1 (6.7)<br>0	 0.326
- **App and Mobile devices** (Secondary function)<br>• *To perform care and assistance tasks – n (%)*	 2 (10.0)	 0 (0)	 0.320

• *To help operators in their work – n (%)*<br>• *To improve self-care – n (%)*<br>• *To have social opportunities – n (%)*<br>• *To stay in communication with others – n (%)*<br>• *To help family members – n (%)*<br>• *No response – n*	2 (10.0)<br>3 (15.0)<br>2 (10.0)<br>9 (45.0)<br>2 (10.0)<br>2	1 (6.7)<br>2 (13.3)<br>6 (40.0)<br>4 (26.7)<br>2 (13.3)<br>0	
- **WS** (Primary function)<br>• *To perform care and assistance tasks – n (%)*<br>• *To help operators in their work – n (%)*<br>• *To improve self-care – n (%)*<br>• *To have social opportunities – n (%)*<br>• *To stay in communication with others – n (%)*<br>• *To help family members – n (%)*<br>• *No response – n*	 1 (5.6)<br>7 (38.9)<br>8 (44.4)<br>0 (0)<br>0 (0)<br>2 (11.1)<br>4	 5 (33.3)<br>2 (13.3)<br>7 (46.7)<br>0 (0)<br>1 (6.7)<br>0 (0)<br>0	 0.081
- **WS** (Secondary function)<br>• *To perform care and assistance tasks – n (%)*<br>• *To help operators in their work – n (%)*<br>• *To improve self-care – n (%)*<br>• *To have social opportunities – n (%)*<br>• *To stay in communication with others – n (%)*<br>• *To help family members – n (%)*<br>• *No response – n*	 2 (11.1)<br>3 (16.7)<br>3 (16.7)<br>1 (5.6)<br>2 (11.1)<br>7 (38.9)<br>4	 5 (33.3)<br>1 (6.7)<br>3 (20.0)<br>2 (13.3)<br>0 (0)<br>4 (26.7)<br>0	 0.391
- **Virtual/Augmented reality** (Primary function)<br>• *To perform care and assistance tasks – n (%)*<br>• *To help operators in their work – n (%)*<br>• *To improve self-care – n (%)*<br>• *To have social opportunities – n (%)*<br>• *To stay in communication with others – n (%)*<br>• *To help family members – n (%)*<br>• *No response – n*	 2 (11.1)<br>7 (38.9)<br>2 (11.1)<br>2 (11.1)<br>3 (16.7)<br>2 (11.1)<br>4	 0 (0)<br>4 (26.7)<br>3 (20.0)<br>4 (26.7)<br>4 (26.7)<br>0 (0)<br>0	 0.347
- **Virtual/Augmented reality** (Secondary function)<br>• *To perform care and assistance tasks – n (%)*<br>• *To help operators in their work – n (%)*<br>• *To improve self-care – n (%)*<br>• *To have social opportunities – n (%)*<br>• *To stay in communication with others – n (%)*<br>• *To help family members – n (%)*<br>• *No response – n*	 6 (33.3)<br>4 (22.2)<br>2 (11.1)<br>4 (22.2)<br>1 (5.6)<br>1 (5.6)<br>4	 2 (13.3)<br>2 (13.3)<br>2 (13.3)<br>3 (20.0)<br>5 (33.3)<br>1 (6.7)<br>0	 0.387

Table S5. Opinions on the usage of support devices, comparing UP and CSS older people feedbacks.

	UP<br>n = 12	CSS<br>n = 10	P value
- **CQ1**<br>***No useful – n (%)***<br>**Yes, low useful – n (%)**<br>***Yes, mildly useful – n (%)***<br>***Yes, very useful – n (%)***	 1 (8.3)<br>0 (0)<br>1 (8.3)<br>10 (83.3)	 0 (0)<br>0 (0)<br>1 (10)<br>9 (90)	 0.645
- **CQ2**<br>***No useful – n (%)***	 0 (0)	 1 (10)	 0.254

Yes, low useful – n (%)<br>*Yes, mildly useful – n (%)*<br>*Yes, very useful – n (%)*	0 (0)<br>2 (16.7)<br>10 (83.3)	1 (10)<br>0 (0)<br>8 (80)	
- **CQ3**<br>*No useful – n (%)*<br>*Yes, low useful – n (%)*<br>*Yes, mildly useful – n (%)*<br>*Yes, very useful – n (%)*	 0 (0)<br>1 (8.3)<br>3 (25.0)<br>8 (66.7)	 0 (0)<br>0 (0)<br>0 (0)<br>10 (100)	 0.130
- **CQ4**<br>*No useful – n (%)*<br>*Yes, low useful – n (%)*<br>*Yes, mildly useful – n (%)*<br>*Yes, very useful – n (%)*	 0 (0)<br>0 (0)<br>4 (33.3)<br>8 (66.7)	 1 (10)<br>0 (0)<br>3 (30)<br>6 (60)	 0.533
- **CQ5**<br>*No useful – n (%)*<br>*Yes, low useful – n (%)*<br>*Yes, mildly useful – n (%)*<br>*Yes, very useful – n (%)*	 0 (0)<br>2 (18.2)<br>1 (9.1)<br>8 (72.7)	 0 (0)<br>1 (10)<br>7 (70)<br>2 (20)	 **0.015**
- **CQ6**<br>*No useful – n (%)*<br>*Yes, low useful – n (%)*<br>*Yes, mildly useful – n (%)*<br>*Yes, very useful – n (%)*	 -	 1 (10)<br>0 (0)<br>1 (10)<br>8 (80)	 -
- **CQ7**<br>*No useful – n (%)*<br>*Yes, low useful – n (%)*<br>*Yes, mildly useful – n (%)*<br>*Yes, very useful – n (%)*	 1 (8.3)<br>3 (25.0)<br>4 (33.3)<br>4 (33.3)	 0 (0)<br>0 (0)<br>1 (10)<br>9 (90)	 0.055
- **CQ8**<br>*No useful – n (%)*<br>*Yes, low useful – n (%)*<br>*Yes, mildly useful – n (%)*<br>*Yes, very useful – n (%)*	 0 (0)<br>1 (8.3)<br>6 (50.0)<br>5 (41.7)	 0 (0)<br>1 (10)<br>1 (10)<br>8 (80)	 0.128
- **CQ9**<br>*No useful – n (%)*<br>*Yes, low useful – n (%)*<br>*Yes, mildly useful – n (%)*<br>*Yes, very useful – n (%)*	 0 (0)<br>0 (0)<br>1 (8.3)<br>11 (91.7)	 0 (0)<br>0 (0)<br>2 (20)<br>8 (80)	 0.427
- **CQ10**<br>*No useful – n (%)*<br>*Yes, low useful – n (%)*<br>*Yes, mildly useful – n (%)*<br>*Yes, very useful – n (%)*	 2 (16.7)<br>2 (16.7)<br>0 (0)<br>8 (66.7)	 0 (0)<br>1 (10)<br>1 (10)<br>8 (80)	 0.365

Table S6. Opinions on the usage of support devices, comparing UP and CSS caregiver feedbacks.			
	UP<br>n = 22	CSS<br>n = 15	P value
- **CQ1**			
No useful – n (%)	0 (0)	0 (0)	0.476
**Yes, low useful – n (%)**	0 (0)	0 (0)	
Yes, mildly useful – n (%)	5 (22.7)	5 (33.3)	
Yes, very useful – n (%)	17 (77.3)	10 (66.7)	
- **CQ2**			
No useful – n (%)	1 (4.5)	0 (0)	**0.030**
Yes, low useful – n (%)	0 (0)	0 (0)	
Yes, mildly useful – n (%)	3 (13.6)	8 (53.3)	
Yes, very useful – n (%)	18 (81.8)	7 (46.7)	
- **CQ3**			
No useful – n (%)	0 (0)	0 (0)	0.259
Yes, low useful – n (%)	0 (0)	0 (0)	
Yes, mildly useful – n (%)	5 (22.7)	6 (40.0)	
Yes, very useful – n (%)	17 (77.3)	9 (60.0)	
- **CQ4**			
No useful – n (%)	0 (0)	0 (0)	0.935
Yes, low useful – n (%)	2 (9.1)	1 (6.7)	
Yes, mildly useful – n (%)	12 (54.5)	9 (60.0)	
Yes, very useful – n (%)	8 (36.4)	5 (33.3)	
- **CQ5**			
No useful – n (%)	1 (4.5)	0 (0)	0.405
Yes, low useful – n (%)	1 (4.5)	3 (20.0)	
Yes, mildly useful – n (%)	7 (31.8)	5 (33.3)	
Yes, very useful – n (%)	13 (59.1)	7 (46.7)	
- **CQ6**			
No useful – n (%)	-	0 (0)	-
Yes, low useful – n (%)		1 (6.7)	
Yes, mildly useful – n (%)		3 (20.0)	
Yes, very useful – n (%)		11 (73.3)	
- **CQ7**			
No useful – n (%)	3 (16.6)	0 (0)	0.322
Yes, low useful – n (%)	0 (0)	0 (0)	
Yes, mildly useful – n (%)	3 (13.6)	2 (13.3)	
Yes, very useful – n (%)	16 (72.7)	13 (86.7)	
- **CQ8**			
No useful – n (%)	0 (0)	0 (0)	0.784
Yes, low useful – n (%)	0 (0)	0 (0)	
Yes, mildly useful – n (%)	5 (22.7)	4 (26.7)	
Yes, very useful – n (%)	17 (77.3)	11 (73.3)	
- **CQ9**			
No useful – n (%)	0 (0)	0 (0)	0.571
Yes, low useful – n (%)	2 (9.1)	1 (6.7)	
Yes, mildly useful – n (%)	4 (18.2)	5 (33.3)	
Yes, very useful – n (%)	16 (72.7)	9 (60.0)	
- **CQ10**			
No useful – n (%)	1 (4.5)	0 (0)	0.231
Yes, low useful – n (%)	0 (0)	2 (13.3)	

Yes, mildly useful – n (%)	10 (45.5)	8 (53.3)	
Yes, very useful – n (%)	11 (50.0)	5 (33.3)	

**Table S7. Possible impact of the PHArA-ON project, comparing UP and CSS older people feedbacks.**			
	UP<br>n = 12	CSS<br>n = 10	P value
- **DQ1**			
No useful – n (%)	0 (0)	0 (0)	0.193
Yes, low useful – n (%)	0 (0)	0 (0)	
Yes, mildly useful – n (%)	4 (33.3)	1 (10)	
Yes, very useful – n (%)	8 (66.7)	9 (90)	
- **DQ2**			
No useful – n (%)	0 (0)	0 (0)	0.255
Yes, low useful – n (%)	1 (8.3)	1 (10)	
Yes, mildly useful – n (%)	0 (0)	2 (20)	
Yes, very useful – n (%)	11 (91.7)	7 (70)	
- **DQ3**			
No useful – n (%)	0 (0)	0 (0)	0.350
Yes, low useful – n (%)	0 (0)	0 (0)	
Yes, mildly useful – n (%)	1 (8.3)	0 (0)	
Yes, very useful – n (%)	11 (91.7)	10 (100)	
- **DQ4**			
No useful – n (%)	0 (0)	0 (0)	0.892
Yes, low useful – n (%)	0 (0)	0 (0)	
Yes, mildly useful – n (%)	1 (8.3)	1 (10)	
Yes, very useful – n (%)	11 (91.7)	9 (90)	
- **DQ5**			
No useful – n (%)	1 (8.3)	2 (20)	0.439
Yes, low useful – n (%)	2 (16.7)	0 (0)	
Yes, mildly useful – n (%)	2 (16.7)	3 (30)	
Yes, very useful – n (%)	7 (58.3)	5 (50)	
- **DQ6**			
No useful – n (%)	1 (8.3)	0 (0)	0.377
Yes, low useful – n (%)	1 (8.3)	0 (0)	
Yes, mildly useful – n (%)	3 (25.0)	1 (10)	
Yes, very useful – n (%)	7 (58.3)	9 (90)	
- **DQ7**			
No useful – n (%)	0 (0)	0 (0)	0.650
Yes, low useful – n (%)	0 (0)	0 (0)	
Yes, mildly useful – n (%)	2 (16.7)	1 (10)	
Yes, very useful – n (%)	10 (83.3)	9 (90)	

Table S8. Possible impact of the PHArA-ON project, comparing UP and CSS caregiver feedbacks.

	UP n = 22	CSS n = 15	P value
- **DQ1**			
No useful – n (%)	0 (0)	0 (0)	0.312
Yes, low useful – n (%)	0 (0)	0 (0)	
Yes, mildly useful – n (%)	6 (27.3)	2 (13.3)	
Yes, very useful – n (%)	16 (72.7)	13 (86.7)	
- **DQ2**			
No useful – n (%)	0 (0)	0 (0)	0.482
Yes, low useful – n (%)	2 (9.1)	0 (0)	
Yes, mildly useful – n (%)	3 (13.6)	2 (13.3)	
Yes, very useful – n (%)	17 (77.3)	13 (86.7)	
- **DQ3**			
No useful – n (%)	1 (4.5)	0 (0)	0.370
Yes, low useful – n (%)	1 (4.5)	0 (0)	
Yes, mildly useful – n (%)	2 (9.1)	4 (26.7)	
Yes, very useful – n (%)	18 (81.8)	11 (73.3)	
- **DQ4**			
No useful – n (%)	0 (0)	0 (0)	0.791
Yes, low useful – n (%)	0 (0)	0 (0)	
Yes, mildly useful – n (%)	2 (9.1)	1 (6.7)	
Yes, very useful – n (%)	20 (90.9)	14 (93.3)	
- **DQ5**			
No useful – n (%)	0 (0)	0 (0)	0.679
Yes, low useful – n (%)	1 (4.5)	0 (0)	
Yes, mildly useful – n (%)	5 (22.7)	3 (20.0)	
Yes, very useful – n (%)	16 (72.7)	12 (80.0)	
- **DQ6**			
No useful – n (%)	2 (9.1)	0 (0)	0.159
Yes, low useful – n (%)	3 (13.6)	0 (0)	
Yes, mildly useful – n (%)	5 (22.7)	2 (13.3)	
Yes, very useful – n (%)	12 (54.5)	13 (86.7)	
- **DQ7**			
No useful – n (%)	1 (4.5)	0 (0)	0.277
Yes, low useful – n (%)	0 (0)	0 (0)	
Yes, mildly useful – n (%)	5 (22.7)	1 (6.7)	
Yes, very useful – n (%)	16 (72.7)	14 (93.3)	

Materials S1. Structured interview performed through videoconference systems or phone calls with older people and informal and formal stakeholders.

SECTION A: Demographic information

Interviewer:	
Date:	
Start:<br>End:	Am/pm<br>Am/pm
User Code	
Pilot site:	☐CSS☐UP
Respondent:	[E] Elder [P] Professional Caregiver [I] InformalCaregiver[S] Service Provider<br>☐01 ☐02 ☐03 ☐04 ☐05 ☐06 ☐07 ☐08 ☐09 ☐ 10
For elderly:	Presence of physical frailty [SI] [NO]<br>Presence of Cognitive frailty [SI] [NO] MMSE score: ______
For professional caregivers and service providers:	How long have you been dealing with the elderly with physical and/or cognitive frailty? (in years) ______
For informalcaregivers:	How long have you been caring for your relative? (in months) _____________<br>How many is the time day care? (in hours) _______________

TELCO	PHONE

SEX:

- ○ Male
- ○ Female

AGE:

- ○ <30
- ○ 30-45
- ○ 46-55
- ○ 55-65
- ○ 65-75
- ○ > 75

SCHOOL EDUCATION:

- ○ Elementary School
- ○ Secondaryschool

- High school
- Graduation

SECTIONB:Use of Technology

Interviewer:	
Date:	
User Code	

Do you frequently use Computer / Smartphone / Tablet? YES NO

If not, why:

____

Do you have family or friends who frequently use Computer / Smartphone / Tablet? YES NO

Which technologies do you consider most interesting based on your needs? Sort from the most interesting (1) to the most marginal (8):

[] Robotics

[] Internet of Things

[] Artificial intelligence / Machine learning

[] Apps and mobile devices

[] Wearables

[] Virtual reality / Augmented reality

Mark in the table a main purpose and a secondary purpose for each proposed technology:

	**Main purpose**	**Secondary purpose**
**Robotics**	[] Care and assistance<br>[] Support to operators<br>[] Self management and well-being<br>[] Sociality<br>[] Communication<br>[] Support to informal carers	[] Care and assistance<br>[] Support to operators<br>[] Self management and well-being<br>[] Sociality<br>[] Communication<br>[] Support to informal carers
**Internet of Things**	[] Care and assistance<br>[] Support to operators<br>[] Self management and well-being<br>[] Sociality<br>[] Communication<br>[] Support to informal carers	[] Care and assistance<br>[] Support to operators<br>[] Self management and well-being<br>[] Sociality<br>[] Communication<br>[] Support to informal carers
**Artificial intelligence / Machine learning**	[] Care and assistance<br>[] Support to operators<br>[] Self management and well-being<br>[] Sociality<br>[] Communication<br>[] Support to informal carers	[] Care and assistance<br>[] Support to operators<br>[] Self management and well-being<br>[] Sociality<br>[] Communication<br>[] Support to informal carers

**Apps and mobile devices**	[] Care and assistance<br>[] Support to operators<br>[] Self management and well-being<br>[] Sociality<br>[] Communication<br>[] Support to informal carers	[] Care and assistance<br>[] Support to operators<br>[] Self management and well-being<br>[] Sociality<br>[] Communication<br>[] Support to informal carers
**Wearables**	[] Care and assistance<br>[] Support to operators<br>[] Self management and well-being<br>[] Sociality<br>[] Communication<br>[] Support to informal carers	[] Care and assistance<br>[] Support to operators<br>[] Self management and well-being<br>[] Sociality<br>[] Communication<br>[] Support to informal carers
**Virtual reality / Augmented reality**	[] Care and assistance<br>[] Support to operators<br>[] Self management and well-being<br>[] Sociality<br>[] Communication<br>[] Support to informal carers	[] Care and assistance<br>[] Support to operators<br>[] Self management and well-being<br>[] Sociality<br>[] Communication<br>[] Support to informal carers

SECTION C: Devices of the support, what I think of the potential of PHArA-ON's services

Interviewer:	
At your place:	
User Code	

**To what extent do you think that the following support devices (robots and / or sensors) could be useful :**	
CQ1. Devices to monitor rest and movements, such as integrated audio-video systems and movement sensors , installed inside the home to reduce the risk of falls	☐ NO, useless<br>☐ YES, not very useful<br>☐ YES, moderately useful<br>☐ YES , very useful
CQ2. Devices for monitoring drug intake, such as pill dispensers and / or scheduled warning systems, to avoid errors in the use of drugs	☐ NO, useless<br>☐ YES, not very useful<br>☐ YES, moderately useful<br>☐ YES , very useful
CQ3. Devices for monitoring environmental conditions (for example, safety systems for controlling temperature, gas-smoke, light, humidity, entry-exit of the main doors, etc.) in order to improve the safety and well-being	☐ NO, useless<br>☐ YES, not very useful<br>☐ YES, moderately useful<br>☐ YES , very useful

CQ4. Devices to regulate heating, humidity, lighting, TV channels	☐ NO, useless<br>☐ YES, not very useful<br>☐ YES, moderately useful<br>☐ YES , very useful
CQ5. Devices for performing a Multidimensional Comprensive Geriatric Assessment	☐ NO, useless<br>☐ YES, not very useful<br>☐ YES, moderately useful<br>☐ YES , very useful
CQ6. Devices for connecting to care programme	☐ NO, useless<br>☐ YES, not very useful<br>☐ YES, moderately useful<br>☐ YES , very useful
CQ7. Devices for monitoring physiological deterioration	☐ NO, useless<br>☐ YES, not very useful<br>☐ YES, moderately useful<br>☐ YES , very useful
CQ8. Devices for monitoring cognitive impairment	☐ NO, useless<br>☐ YES, not very useful<br>☐ YES, moderately useful<br>☐ YES , very useful
CQ9. Devices for connecting with friends and family	☐ NO, useless<br>☐ YES, not very useful<br>☐ YES, moderately useful<br>☐ YES , very useful
CQ10. Bus / taxi service to promote the mobility of the elderly within the city	☐ NO, useless<br>☐ YES, not very useful<br>☐ YES, moderately useful<br>☐ YES , very useful

Description of the project

General description of the PHArA-ON project

PHArA-ON : Pilots for healthy and active aging

PHArA-ON (Pilots forHealthy and Active Aging) is an innovation project funded by the European Union's Horizon 2020 program. This large-scale pilot project involves partners from 12 European countries and aims to improve the quality of life of the elderly European population.

PHArA-ON will create a series of highly customizable interoperable open platforms, which will integrate advanced services, devices and tools including IoT, artificial intelligence, robotics, cloudcomputing, smart wearable devices, big data and intelligent analysis. These solutions will be extensively tested and validated, with the aim of responding to the needs of the elderly and improving their independence, safety and skills.

PHArA-ON aims to find solutions for European industry and policy makers to successfully face the challenges of aging populations in the coming years. In addition, it is proposed to develop new collaborative partnerships between actors from different sectors to ensure that the platforms meet the needs of the elderly and formal and informal caregivers. At the same time, PHArA-ON offers digital instrument suppliers the opportunity to use their devices and systems as a support for an increasingly older population.

The project is a collaboration of 41 organizations, led by the SSSA (Sant'Anna School of Advanced Studies and Specialization, based in Pisa, Italy) and will last 48 months (from December 2019 to November 2023). PHArA-ON adopts a user-centered approach and will test different digital solutions in 6 different pilots in 5 countries: Italy (Tuscany-Puglia), Spain (Murcia and Andalusia), the Netherlands (Twente), Slovenia (Island) and Portugal (Coimbra-Amadora).

PHArA-ON's partners include large, medium and small businesses, research organizations, universities, health authorities, public and private health care providers, social organizations, health institutes and standardization bodies. With an investment of around € 21 million, PHArA-ON has the ambition to make a significant contribution to the European Union's agenda on active and healthy aging.

The PHArA-ON partners come from Italy, Spain, Portugal, the Netherlands, Slovenia, Croatia, Germany, France, the United Kingdom, Belgium, Austria, Estonia.

Contacts:

Website: www.PHArA-ON .eu
Facebook: fb.me/ PHArA-ON .project
Twitter: @ PHArA-ON Project
LinkedIn: PHArA-ON - Pilots for Healthy and Active Aging https://www.linkedin.com/groups/12335464/

Interview via Skype: Slide presentation of the Services

During the interview, PHArA-ON's services will be presented through a PPT

Grazie a Pharaon sarà possibile…

- Utilizzare la TV per comunicare con parenti o amici.
- Entrare in contatto con altri anziani che abitano in zona, grazie all'aiuto di un animatore.
- Visionare, dalla propria TV, filmati su argomenti di interesse e partecipare ad attività di gruppo sia reali che virtuali: giochi, conversazioni, visione collettiva di video.
- Attraverso la TV , fare delle attività che allenano la memoria e migliorano le competenze cognitive.

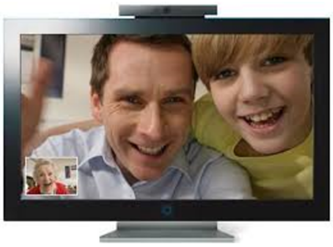

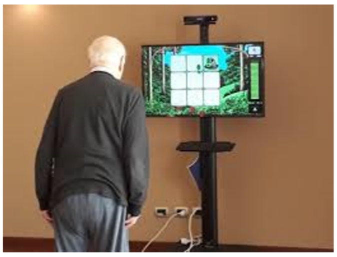

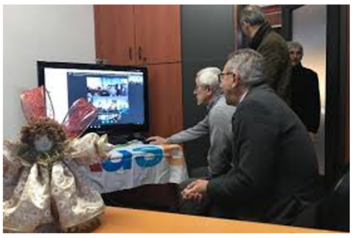

2

Interview via phone: Service story

During the telephone interview it will not be possible to show the power point presentation. For this reason, the services will be presented narratively to the interlocutor.

CSS - Apulian

Mario is an 80-year-old man who lives alone in a municipality in the province of Foggia .

He has a 48-year-old daughter, married, who lives in another municipality, about 20 km from him.

Mario does not have many relationships with the neighborhood, he visits the country's shops sporadically, because he moves less and less easily, and for this he feels lonely and sometimes sad. Mario has also had some memory problems for some time, and this scares him.

Thanks to PHArA-ON Mario could use his TV to communicate with his daughter and other relatives or friends.

A social worker could visit him weekly and put him in contact with other elderly people who live in his area. The animator could send him, again through television, films on topics that interest him and could organize both real and virtual group activities: games, conversations, collective viewing of videos.

Through TV, Mario could also do activities that train his memory and improve his cognitive skills.

At Mario's home, a simple robot could be used which, guided remotely, would allow an operator to quickly communicate with Mario and perform video surveillance activities.

Some sensors installed in his home could monitor his movements, and report to his daughter or the operator of reference if there is something abnormal: no movement, door or windows open, lights on at night, etc.

Mario could wear a bracelet (or a ring) that detects some vital signs and monitors his physical activity. Furthermore, Mario could carry out a Multidimensional Evaluation which allows to examine the clinical, functional, cognitive, nutritional and social aspects of the elderly person .

A collective transport service, managed by volunteers, could be set up which Mario and his friends could use to run errands and other outings in the area, bookable through TV .

All the information gathered from the activities carried out with the TV, the sensors and the bracelet could flow into a single platform, which by reworking them generates new indications to improve Mario's quality of life: these indications could be communicated to Mario through TV.

The medical staff (doctor, nurse, obs ...) who follows Mario could access the platform, in order to customize the treatment based on the information received.

Even Mario's daughter could access the platform through her smartphone, making sure that the father is well, communicating with him and intervening in case of need. Through the smartphone, it could also receive psychological support and advice from qualified personnel, should the load of care reveal its need.

UP - Tuscany

Mario is an 80-year-old man who lives alone in a municipality in the province of Florence.

He has a 48-year-old daughter, married, who lives in another municipality, about 20 km from him.

Mario does not have many relationships with the neighborhood, he visits the country's shops sporadically, because he moves less and less easily, and for this he feels lonely and sometimes sad. Mario has also had some memory problems for some time, and this scares him.

Thanks to PHArA-ON Mario could use his TV to communicate with his daughter and other relatives or friends.

A social worker could visit him weekly and put him in contact with other elderly people who live in his area. The animator could send him, again through television, films on topics that interest him and could organize both real and virtual group activities: games, conversations, collective viewing of videos.

Through TV, Mario could also do activities that train his memory and improve his cognitive skills.

A collective transport service, managed by volunteers, could be set up which Mario and his friends could use to run errands and other outings in the area, bookable through TV.

Some sensors installed in his home could monitor his movements, and report to his daughter or the operator of reference if there is something abnormal: no movement, door or windows open, lights on at night, etc.

Mario could wear a bracelet (or a ring) that detects some vital signs and monitors his physical activity.

All the information gathered from the activities carried out with the TV, the sensors and the bracelet could flow into a single platform, which by reworking them generates new indications to improve Mario's quality of life: these indications could be communicated to Mario through TV.

The medical staff (doctor, nurse, obs ...) who follows Mario could access the platform, in order to customize the treatment based on the information received.

Even Mario's daughter could access the platform through her smartphone, making sure that the father is well, communicating with him and intervening in case of need. Through the smartphone, it could also receive psychological support and advice from qualified personnel, should the load of care reveal its need.

SECTION D: Impact of PHArA-ON

Interviewer:	
At your place:	
User Code	

**To what extent do you think PHArA-ON can be useful for:**	
DQ1. Improve the quality of life	☐ NO, useless<br>☐ YES, not very useful<br>☐ YES, moderately useful<br>☐ YES , very useful
DQ2. Improve the quality of care	☐ NO, useless<br>☐ YES, not very useful<br>☐ YES, moderately useful<br>☐ YES , very useful
DQ3. Improve safety in daily life activities	☐ NO, useless<br>☐ YES, not very useful<br>☐ YES, moderately useful<br>☐ YES , very useful
DQ4. Send emergency alert / communication messages	☐ NO, useless<br>☐ YES, not very useful<br>☐ YES, moderately useful<br>☐ YES , very useful
DQ5. Improve the assistance provided with home physical and cognitive rehabilitation programs	☐ NO, useless<br>☐ YES, not very useful<br>☐ YES, moderately useful

	☐ YES , very useful
DQ6. Detect when a person is becoming more lonely and isolated	☐ NO, useless<br>☐ YES, not very useful<br>☐ YES, moderately useful<br>☐ YES , very useful
DQ7. Track changes in health status	☐ NO, useless<br>☐ YES, not very useful<br>☐ YES, moderately useful<br>☐ YES , very useful

8. (For formal, informal and service providers only) What additional services do you think PHArA-ON should have to increase functional autonomy in the elderly?

____
____
____

SECTION E: Users' needs and priorities

Interviewer:	
Date:	
User Code	

[Please refer to the interview methodology for conversation stimulation questions]

1. Considering all the activities that are performed daily to help an elderly person, what activities could be performed by the patient with the help of a robot or sensors?

____
____
____

2. What does a robot need in order to help an elderly person perform the tasks listed above?

____
____
____

3. If a new person would like to offer assistance to an elderly person, what is it good to get to know him or her in order to make him or her better care?

____
____
____

4. People often report that patients who develop cognitive and/or physical deficits become lonely and tend to isolate themselves: is this what you are experiencing or observing while caring for your family member/patient? If so, what do you think might help to stay in touch with friends and family?

____
____
____

5. What are the barriers that prevent you or your family member/patient from staying in touch with the community and friends?

____
____
____

6. What do you think about using an assistant robot to help people with cognitive and/or physical impairments overcome isolation and loneliness by keeping them better in touch with others?

____

7. The PHArA.ON system, which we are developing, will be able to collect information about people's condition through simple questions and the use of sensors. What do you think about it?

8. What kind of information about people's condition could be important to collect?

11. What could be the upside if an elderly person with cognitive and/or physical impairment had an assistant robot?

12. Is it likely there are no upsides? What potential problems could there be if an elderly person with cognitive and/or physical impairment had an assistant robot? Suggestions: any concerns? Some doubts about replacing human support with an assistant robot? Some doubts about personal data protection?

13. What other advice would you give us for the development of the PHArA.ON System?

SECTION F: Conclusions and notes on the interview

Interviewer:	
Date:	
User Code	

[On this page all the other notes to be collected during the interview are rewritten]
